# Autonomic dysregulation and nonsuicidal self-injury: findings from a cross-sectional online survey

**DOI:** 10.3389/fpsyt.2026.1874303

**Published:** 2026-07-27

**Authors:** Andreas Goreis, Jacqueline Alsch, Sofia-Marie Oehlke, Paul L. Plener, Oswald D. Kothgassner

**Affiliations:** 1 Department of Child and Adolescent Psychiatry, Medical University of Vienna, Vienna, Austria; 2 Comprehensive Center for Pediatrics (CCP), Medical University of Vienna, Vienna, Austria; 3 Comprehensive Center for Clinical Neuroscience and Mental Health (C3NMH), Medical University of Vienna, Vienna, Austria; 4 Department of Child and Adolescent Psychiatry and Psychotherapy, University of Ulm, Ulm, Germany

**Keywords:** autonomic dysregulation, autonomic nervous system, children and adolescents, nonsuicidal self-injury, online survey

## Abstract

**Introduction:**

Dysregulation of the autonomic nervous system has been implicated in nonsuicidal self-injury (NSSI) in laboratory studies of physiological reactivity, but it remains unclear whether self-reported everyday autonomic symptom burden is associated with NSSI severity.

**Methods:**

We conducted a preregistered cross-sectional online survey of N = 1,002 German-speaking adolescents and young adults aged 14–30 years (M = 23.43; 73% women). Autonomic symptoms were assessed with the COMPASS 31; NSSI presence and frequency were measured with the SITBI-R across four reference periods (past week, month, year, lifetime). Data were analyzed using zero-inflated negative binomial models.

**Results:**

The 12-month NSSI prevalence was 22.6%. Higher COMPASS 31 scores were associated with lower odds of reporting zero NSSI episodes across all reference periods and with higher NSSI episode counts for the past month, past year, and lifetime; the past-week count association was not statistically significant. These associations held after controlling for childhood adversity, perceived stress, medication, and hormonal contraception.

**Discussion:**

These results indicate that self-reported autonomic symptom burden was robustly associated with NSSI involvement, particularly with the likelihood of any NSSI and with episode counts over longer reference periods. These findings suggest that self-reported autonomic symptom burden may represent a clinically relevant somatic correlate of NSSI severity.

## 1 Introduction

Nonsuicidal Self-Injury (NSSI) is the deliberate, repeated infliction of bodily harm without suicidal intent—typically cutting, scratching, burning, or hitting oneself (1). NSSI disorder (NSSI-D) is listed in Section III of the DSM-5 as a condition warranting further study, defined as self-harm on at least five days within the past year (2). A systematic review of longitudinal studies (3) found that NSSI prevalence peaks at ages 15–16 and declines by age 18. Nevertheless, the behavior remains common, with global prevalence estimates of 16–22% among adolescents (4, 5), and roughly 13% among young adults (6), although data for the latter group are more limited. Importantly, NSSI is not restricted to adolescence, as prior work suggests that NSSI may show a second period of elevated occurrence in emerging adulthood, particularly around the transition to college or university (7, 8). NSSI is also a significant predictor of later suicide attempts (9); for example, Voss et al. (10) found that two-thirds of individuals who had attempted suicide had previously engaged in NSSI.

Clinically, NSSI is seldom isolated; most individuals also meet criteria for depression, anxiety, PTSD, eating disorders, ADHD, or personality disorders (11–13). Reflecting this transdiagnostic pattern, Lengel et al. (14) have recently proposed reclassifying NSSI as a clinical specifier rather than a standalone diagnosis to capture its heterogeneity and dimensional nature. Irrespective of diagnostic classification, NSSI is most commonly employed to modulate overwhelming emotion or stress (15, 16), a function that makes the behavior clinically pressing and highlights the need to understand its underlying mechanisms.

Although social and psychological factors associated with NSSI are well studied, and several psychological interventions show promise for reducing adolescent self-harm and related outcomes (17, 18), the evidence base for interventions specifically targeting NSSI remains comparatively limited. Research on biological underpinnings is still in its infancy. Current pathophysiological models of NSSI (19) separate distal biological traits—including genetic and epigenetic risk and adverse childhood experiences (ACEs)—from proximal biological traits, such as alterations in brain structure or function and dysregulated stress-response systems. Accumulating evidence shows that exposure to acute or chronic stressors—especially ACEs—heightens the likelihood of NSSI, a link thought to operate through subsequent alterations in neuroendocrine and autonomic stress-response systems (20). Experimental work likewise points to dysregulation in the body’s two chief stress systems—the autonomic nervous system (ANS) and the hypothalamic-pituitary-adrenal (HPA) axis—among individuals who self-injure, although the precise role these systems play in everyday NSSI remains unclear (see, 21, for meta-analyses).

The ANS, which continually balances parasympathetic “rest-and-digest” and sympathetic “fight-or-flight” activity to modulate organ function (22), has become a compelling biomarker candidate for NSSI because it represents the brain–body circuitry involved in emotion regulation and inhibitory control (23, 24). According to the Neurovisceral Integration Model (25, 26), a cortico-subcortical “central autonomic network”—including the prefrontal and anterior cingulate cortices, amygdala, and hypothalamus—coordinates sympathetic-parasympathetic balance and links cognitive, emotional, and physiological control to adaptive behavior. Within this framework, autonomic dysfunction may be relevant to NSSI severity because reduced autonomic flexibility can intensify or prolong aversive bodily-emotional states. For individuals who use NSSI to regulate affect or stress, repeated reliance on self-injury may therefore become more likely when stress-related arousal is difficult to downregulate, providing a plausible pathway from stress to heightened, affect-regulatory self-injury.

Beyond laboratory stress tasks that capture phasic reactivity (21), resting-state and ambulatory studies also suggest altered autonomic regulation in individuals who engage in NSSI. For example, reduced vagally mediated heart-rate variability has been reported in borderline personality disorder, a condition frequently characterized by affective instability, impulsivity, and self-harm (27), and flatter diurnal trajectories of heart rate and heart-rate variability have been observed in adolescents meeting criteria for NSSI disorder (28). These physiological findings support the relevance of autonomic regulation for models of NSSI (19), but they do not address whether young people also experience everyday bodily symptoms that map onto autonomic symptom burden. This distinction is important because self-reported autonomic symptoms, such as orthostatic dizziness, vasomotor complaints, gastrointestinal symptoms, bladder symptoms, or pupillomotor complaints, capture the subjective bodily problems that may be noticed in daily life and reported in clinical or survey contexts. Conceptually, the construct of autonomic symptom burden differs from measures of anxiety, somatization, or general psychological distress because it reflects symptom domains commonly linked to autonomic functioning rather than affective or cognitive distress symptoms. However, these constructs may overlap, as psychological distress is often accompanied by bodily symptoms. Accordingly, autonomic symptom measures should not be treated as substitutes for, e.g., physiological ANS assessment, nor can they establish autonomic mechanisms. They may, however, help characterize a clinically accessible somatic symptom dimension that co-occurs with NSSI severity, but whether self-reported autonomic symptom burden is associated with NSSI frequency—especially during adolescence and emerging adulthood, where NSSI is common (6)—remains to be elucidated.

To our knowledge, prior work has not evaluated whether self-reported autonomic symptom burden is related to NSSI frequency. In this study, we therefore asked whether self-reported autonomic symptoms, measured via a validated clinical instrument, the COMPASS 31 (29), were associated with NSSI frequency across four reference periods (past week, month, year, lifetime) in a large sample of 14- to 30-year-olds. To evaluate whether this association extended beyond established stress-related correlates of NSSI, we additionally adjusted for ACEs and perceived stress (28, 30). Because NSSI frequency is typically characterized by many zero responses and substantial overdispersion, we used zero-inflated negative binomial models to distinguish between two related but conceptually different outcomes: the odds of reporting no NSSI episodes and the expected number of episodes among those at risk for NSSI. We tested one overarching preregistered hypothesis across four clinically relevant reference periods: higher self-reported autonomic symptom burden would be associated with lower odds of zero NSSI episodes and with higher NSSI episode counts across the past week, past month, past year, and lifetime.

## 2 Methods

### 2.1 Participants and procedure

The study was performed in accordance with the Declaration of Helsinki. The study was approved by the institutional review board of the Medical University of Vienna (#1794/2024), and participants provided online informed consent prior to participation. Recruitment was conducted between October 2024 and January 2025 via social-media advertisements, university mailing lists, and mental-health forums; the survey itself was hosted on SoSciSurvey. Recruitment materials explicitly referred to the study topic as “autonomic nervous system and self-injury”. No monetary or material incentives were offered. No instructed-response attention checks or technical verification checks were embedded in the survey. To preserve anonymity and comply with data protection requirements, IP addresses were not stored. Browser-based cookie tracking was enabled in SoSciSurvey to reduce repeated participation from the same browser. We relied on a convenience, *ad hoc* sample of self-selected respondents rather than a stratified or population-representative cohort. Eligibility criteria were (a) sufficient command of German and (b) age between 14 and 30 years. Of 2,992 individuals who accessed the survey link, 1,283 exited before completing any questionnaires and 1,004 finished the survey. Two cases were removed because the key NSSI-frequency items were missing, yielding a final sample of *N* = 1,002. The survey was configured to alert participants to missing responses before they could proceed to the next page, resulting in complete item-level data among those 1,002 participants.

### 2.2 Measures

First, we collected sociodemographic information (gender, age). Participants then completed two yes/no items: one on medication use (“Are you currently taking, or have you taken, any medication within the past month?”) and one on hormonal contraception (“Are you currently using, or have you used, hormonal contraceptive methods in the past month? This includes, for example, the birth-control pill, hormonal IUD, contraceptive patch, or contraceptive injection; copper or gold IUDs are not included.”). All remaining self-report instruments were administered in random order.

#### 2.2.1 Autonomic symptoms

We administered the 31-item Composite Autonomic Symptom Score (COMPASS 31) (29) to assess self-reported autonomic symptom burden across six domains: orthostatic intolerance, vasomotor, secretomotor, gastrointestinal, bladder, and pupillomotor symptoms. The questionnaire asks about everyday bodily symptoms that are commonly linked to autonomic functioning, including dizziness or lightheadedness when standing, sweating abnormalities, gastrointestinal complaints, bladder symptoms, and visual symptoms related to pupil regulation. The COMPASS 31 was originally developed as a refined and abbreviated self-report measure for autonomic symptom assessment in clinical autonomic disorders and neurological conditions (29). It has since been used in behavioral and medical research contexts in which autonomic complaints are clinically relevant, including post-acute sequelae of COVID-19 (31). Domain scores are weighted and summed to a total score ranging from 0 to 100, with higher scores indicating greater self-reported autonomic symptom burden. In the present sample, internal consistency was good (McDonald’s ω = .87).

#### 2.2.2 Nonsuicidal self-injury

Items from the German version of the revised Self-Injurious Thoughts and Behaviors Interview (SITBI-R) (32) were administered to assess the presence, frequency, methods, and characteristics of NSSI. The SITBI-R is a structured assessment of self-injurious thoughts and behaviors with evidence for reliability and validity across self-harm-related outcomes (32). Participants were first asked whether they had ever intentionally injured themselves without wanting to die, accompanied by examples of NSSI methods such as cutting, scratching, burning, biting, hitting oneself, or inserting objects under the skin or nails. If participants endorsed lifetime NSSI, they were asked to report NSSI frequency across four reference periods: lifetime, past 12 months, past month, and past week. Frequency responses were entered as open numeric response fields. For example, participants indicated how often they had injured themselves during the past month. These count variables served as the primary outcomes in the zero-inflated negative binomial models.

#### 2.2.3 Adverse childhood experiences

We administered the German version of the Childhood Trauma Questionnaire (CTQ) (33) to assess ACEs. The CTQ comprises 25 clinical items rated on a 5-point scale ranging from 1 = “never true” to 5 = “very often true” and assesses maltreatment during childhood and adolescence across five domains: emotional abuse, physical abuse, sexual abuse, emotional neglect, and physical neglect. Items ask, for example, about experiences of emotional humiliation, physical harm, sexual boundary violations, insufficient emotional support, or inadequate physical care during childhood. The German version has shown satisfactory psychometric properties in a representative population sample (33). Internal consistency in the present sample was excellent (McDonald’s ω = .95).

#### 2.2.4 Perceived stress

Perceived stress was assessed with the German version of the 10-item Perceived Stress Scale (PSS-10) (34). The PSS-10 assesses the extent to which respondents experienced their life as unpredictable, uncontrollable, and overloaded during the previous month. Items ask, for example, how often participants felt unable to control important things in their life or felt that difficulties were accumulating. The German version has shown good psychometric properties in a representative German community sample (32). Responses are given on a 5-point scale from 0 = “never” to 4 = “very often,” with higher total scores indicating greater perceived stress. Internal consistency in the present sample was high (McDonald’s ω = .90).

### 2.3 Analyses

Hypotheses were tested with zero-inflated negative binomial (ZINB) models in R (version 4.4.2) using the pscl package, chosen to accommodate overdispersion and the excess of zero counts in the NSSI frequency outcomes. Eight preregistered models (see https://doi.org/10.17605/OSF.IO/ZD5CG) were estimated: four unadjusted models examining associations between COMPASS 31 total scores and NSSI frequency for the past week, past month, past year, and lifetime, followed by four adjusted models that additionally included ACEs (CTQ total score), perceived stress (PSS-10 total score), medication use (yes/no), and hormonal contraception use (yes/no) as covariates. As a non-preregistered exploratory sensitivity analysis, we additionally repeated the adjusted models with gender included alongside the original covariates. In ZINB models, exponentiated coefficients from the count component are incidence-rate ratios (IRRs), with values above 1 indicating higher expected NSSI episode counts. Exponentiated coefficients from the zero-inflation component are odds ratios (ORs), with values below 1 indicating lower odds of belonging to the always-zero group, that is, lower odds of reporting no NSSI episodes. All analytic data and code are available on the Open Science Framework (doi: 10.17605/OSF.IO/NEJPQ).

## 3 Results

Of the 1,002 people (mean age: 23.43, *SD* = 3.72) who participated in our study, 483 (48.2%) reported having engaged in NSSI at least once in their lifetime. Among these, 377 (78.1%) identified as women, 81 (16.8%) as men, and 25 (5.2%) as diverse. The mean age at first NSSI was 14.20 years (*SD* = 3.17). Among individuals who reported lifetime NSSI—and allowing for multiple responses—the most frequently reported methods were cutting/carving (71.4%), scratching (54.2%), hitting oneself (44.3%), biting (32.1%), burning (19.9%), “other” methods (10.8%), and inserting objects under the skin or nails (7.2%). The 12-month NSSI prevalence was 22.6%. Using the DSM-5 frequency criterion of at least five NSSI days in the past year, prevalence was 11.0%. Descriptive statistics for the questionnaires are presented in **Table 1**.

**Table 1:** Sample characteristics.

Characteristic	Total sample (*N* = 1,002)	Woman (*N* = 729)	Man (*N* = 234)	Diverse (*N* = 37)	*F* (ANOVA)
	*M* (*SD*)	*M* (*SD*)	*M* (*SD*)	*M* (*SD*)	
Age	23.43 (3.72)	23.38 (3.58)	23.58 (4.12)	23.37 (3.83)	0.297
NSSI last week	0.18 (1.45)	0.20 (1.66)	0.13 (0.63)	0.08 (0.28)	0.297
NSSI last month	0.74 (6.63)	0.82 (7.64)	0.53 (2.41)	0.65 (2.16)	0.182
NSSI last year	6.45 (67.79)	7.14 (78.14)	4.69 (25.36)	4.27 (9.82)	0.135
NSSI lifetime	37.46 (185.02)	36.56 (184.83)	33.17 (184.46)	84.30 (195.81)	1.255
Autonomic symptom burden (COMPASS 31)	21.14 (13.84)	22.45 (13.58)	16.97 (13.56)	21.94 (15.54)	14.31***
Adverse childhood experiences (CTQ)	40.06 (13.91)	39.28 (13.13)	39.75 (13.20)	57.92 (20.48)	33.75***
Perceived stress (PSS-10)	20.36 (7.28)	20.85 (7.15)	18.74 (7.40)	21.27 (7.81)	7.845***
Medication intake (yes)	360 (35.93%)	271 (37.17%)	74 (31.26%)	15 (40.54%)	–
Hormonal contraception (yes)	261 (26.06%)	253 (34.71%)	3 (1.28%)	4 (10.81%)	–

***denotes statistically significant differences (*p* <.001).

Women scored higher than men on self-reported autonomic symptom burden (COMPASS 31; mean difference = 5.48, 95% CI [3.07, 7.88], *p* <.001). For ACEs (i.e., the CTQ), participants identifying as diverse scored higher than both men (mean difference = 18.46, 95% CI [13.31, 23.98], *p* <.001) and women (mean difference = 18.17, 95% CI [12.57, 23.77], *p* <.001). Perceived stress (PSS-10) was likewise elevated in women relative to men (mean difference = 2.11, 95% CI [0.84, 3.89], *p* <.001). Across the total sample, ACEs were modestly and positively correlated with self-reported autonomic symptom burden (*r* = .26, 95% CI [.20,.31], *p* <.001). The medication classes reported most frequently were, in descending order, analgesics, antidepressants, antibiotics, thyroid hormones, antipsychotics, antihistamines, and psychostimulants. Among those who had taken medication during the past month, 109 participants used a single drug, whereas 241 used two or more.

### 3.1 Association between self-reported autonomic symptom burden and NSSI

**Figure 1:**
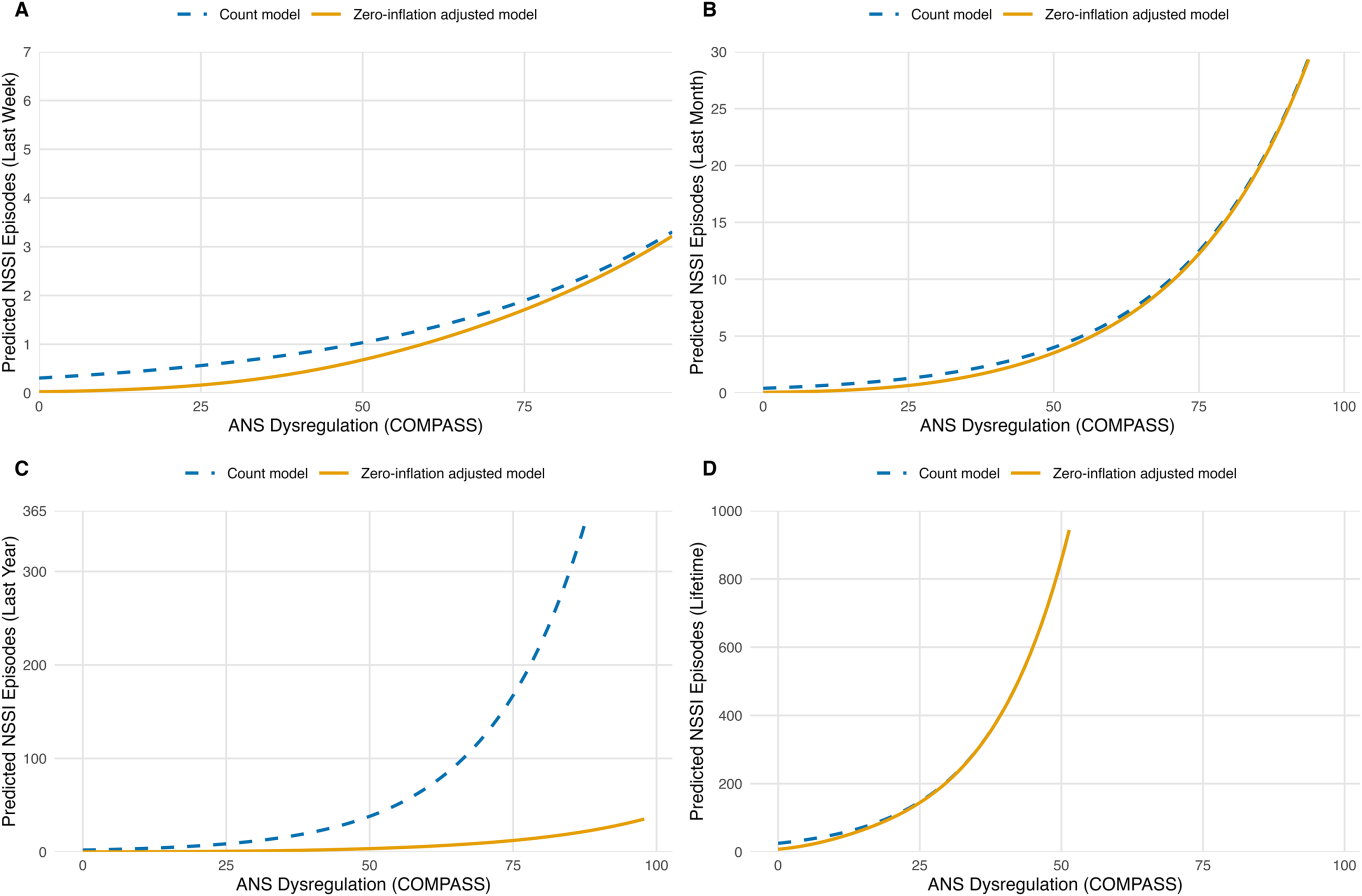
Model-predicted NSSI frequency by self-reported autonomic symptom burden. Predicted episode counts are based on the preregistered unadjusted zero-inflated negative binomial models with COMPASS 31 total score as the focal predictor. Panels show NSSI frequency over **(A)** the past week, **(B)** the past month, **(C)** the past year, and **(D)** lifetime.

A series of preregistered zero-inflated negative binomial regression models showed that, in the past-week model, a one-point increase on the COMPASS 31 was not significantly associated with a higher episode count (IRR = 1.02, 95% CI [0.99, 1.06], *p* = .17), but was associated with lower odds of reporting zero NSSI episodes (OR = 0.94, 95% CI [0.91, 0.97], *p* <.001). In the past-month model, each additional COMPASS 31 point was associated with a 5% increase in expected episodes (IRR = 1.05, 95% CI [1.01, 1.08], *p* = .010) and lower odds of zero episodes (OR = 0.93, 95% CI [0.89, 0.97], *p* <.001). The past-year model showed a 6% increase in expected episode count (IRR = 1.06, 95% CI [1.03, 1.09], *p* <.001) and lower zero-episode odds (OR = 0.92, 95% CI [0.89, 0.95], *p* <.001) per COMPASS 31 point. In the lifetime model, each COMPASS 31 point was associated with a 7% higher cumulative episode count (IRR = 1.07, 95% CI [1.05, 1.10], *p* <.001) and lower odds of never having engaged in NSSI (OR = 0.82, 95% CI [0.73, 0.93], *p* = .002). Dispersion parameters indicated substantial overdispersion, supporting the negative binomial specification (see **Figures 1A–D**).

### 3.2 Covariate model

When ACEs, perceived stress, medication use, and hormonal contraception use were added as covariates, the main COMPASS 31 associations were retained. In the count component, each additional COMPASS 31 point was associated with higher NSSI episode counts for the past month (IRR = 1.05, *p* = .002), past year (IRR = 1.05, *p* <.001), and lifetime (IRR = 1.03, *p* <.001), whereas the past-week count association remained non-significant (IRR = 1.03, *p* = .08). In the zero-inflation component, higher COMPASS 31 scores were associated with lower odds of reporting zero NSSI episodes in all four models. Perceived stress was independently associated with higher episode counts in the past-week, past-month, and past-year models. CTQ scores showed a mixed pattern: higher CTQ scores were inversely associated with past-month and past-year episode counts but positively associated with lifetime counts, while also being associated with lower odds of zero episodes in the past-month and past-year models. The inverse CTQ associations in the month and year count components should be interpreted cautiously and may reflect statistical suppression or shared variance with perceived stress and COMPASS 31 scores, rather than a protective effect of childhood adversity.

Medication use and hormonal contraception were not consistently associated with NSSI outcomes across models. Dispersion parameters remained indicative of substantial overdispersion, supporting the negative binomial specification. In non-preregistered exploratory sensitivity models additionally adjusting for gender, the COMPASS 31 associations were largely retained, with significant count-component associations across all four reference periods and significant zero-inflation associations for past-year and lifetime NSSI. Full coefficient estimates for the unadjusted, covariate-adjusted, and exploratory gender-adjusted models are provided in **Supplementary Tables 1**–**3**.

## 4 Discussion

In this preregistered cross-sectional survey of German-speaking adolescents and young adults, higher self-reported autonomic symptom burden was associated with NSSI frequency across several reference periods. This finding extends prior laboratory-based work on physiological autonomic regulation and NSSI (21) by showing that the association is also reflected at the level of everyday bodily symptoms reported by participants. The present study therefore does not establish autonomic symptoms as causal predictors of NSSI, nor does it show that self-report measures can replace physiological assessment. Rather, it identifies self-reported autonomic symptom burden as a clinically accessible somatic correlate of NSSI severity. Practically, this suggests that autonomic symptom burden may be useful to assess alongside NSSI, not as a proxy for NSSI itself, but as a complementary indicator of bodily distress and stress-related dysregulation. In clinical contexts, elevated autonomic symptoms in young people reporting NSSI may therefore inform case formulation and prompt further assessment of somatic complaints, stress sensitivity, and emotion-regulation difficulties.

The pattern of effects across our preregistered analyses yields several observations. First, higher autonomic-symptom scores consistently lowered the odds of reporting zero NSSI episodes, even in the one-week window, indicating that self-reported autonomic symptom burden is associated with the occurrence of recent self-injury. Second, associations with episode counts were evident for the past month, past year, and lifetime, whereas the past-week count component was not statistically significant. This pattern suggests that autonomic symptom burden may be more closely related to broader NSSI involvement and cumulative severity than to very short-term fluctuations in episode counts. Although the per-point effects were modest, they may be clinically meaningful across larger differences in symptom burden: for example, a 10-point difference in COMPASS 31 scores corresponds to approximately 34–63% higher expected NSSI counts in the significant adjusted count models. The stronger associations across longer reference periods are broadly consistent with physiological findings indicating that chronic autonomic inflexibility, including reduced vagal tone and altered sympathetic regulation, is particularly relevant in individuals with persistent NSSI (21, 28). Third, whereas few studies have linked graded levels of NSSI to physiological indices (35, 36), our findings suggest that self-reported autonomic symptom burden may track a related severity continuum at the level of everyday bodily symptoms. Given that higher NSSI frequency is associated with later suicide attempts and broader psychopathology (37, 38), this somatic symptom dimension may be clinically meaningful. However, because the data are cross-sectional and the NSSI time windows were assessed retrospectively, the observed gradient should not be interpreted as evidence that autonomic symptoms drive escalation of NSSI over time. Rather, the findings indicate that young people reporting greater autonomic symptom burden also tend to report greater NSSI involvement, particularly when NSSI is assessed over longer reference periods. Clinically, this pattern may help identify individuals whose self-injury occurs in the context of broader bodily distress and stress-regulation difficulties, which may be relevant for assessment and treatment planning.

We also found that the associations between COMPASS 31 scores and NSSI were retained after adjustment for ACEs, perceived stress, medication use, and hormonal contraception, suggesting that self-reported autonomic symptom burden captures variance in NSSI involvement that is not fully accounted for by these established stress-related and health-related covariates (39). This supports the value of assessing everyday autonomic symptoms as a clinically accessible dimension of bodily dysregulation in the context of NSSI, particularly because such symptoms may reflect how stress-related physiological vulnerability is experienced in daily life. In our models, perceived stress was independently associated with higher episode counts in the past-week, past-month, and past-year models, whereas CTQ scores showed a more complex pattern: they were inversely associated with month- and year-counts but positively associated with lifetime counts. This finding should be interpreted cautiously. One possible explanation is that childhood adversity is more strongly related to cumulative lifetime vulnerability for NSSI, whereas more recent NSSI frequency may be more closely tied to current perceived stress, current autonomic symptom burden, or other unmeasured clinical states. This interpretation is broadly compatible with developmental frameworks that conceptualize ACEs as distal liabilities and stress-regulatory alterations as more proximal correlates of self-injury (19), as well as evidence linking childhood adversity to long-term alterations in HPA axis and autonomic functioning (40). Alternatively, the inverse CTQ associations in the month and year count models may reflect statistical suppression or model instability due to shared variance between CTQ, perceived stress, and COMPASS 31 scores. Thus, although the cross-sectional design precludes conclusions about temporal ordering, mediation, or causal pathways, the present findings support the incremental descriptive and clinical value of measuring everyday autonomic symptom burden when characterizing NSSI severity.

The 12-month prevalence of NSSI in our sample was 22.6%, roughly twice the 10–12% reported in recent meta-analyses of community studies (4, 5), and lifetime prevalence reached 48%. However, comparable figures (at least regarding the 12-month prevalence) have been observed in earlier work conducted in Austria: Plener et al. (41) reported 11% within six months, and Brunner et al. (42) found 27% lifetime. Several design features may have contributed to the elevated estimates: recruitment via social media, university lists, and mental-health forums; full anonymity; and an online SITBI-R version that probed NSSI methods across four time frames. While such detail improves recall, it may also prompt respondents to classify minor injuries as NSSI, especially for lifetime reports (see Hooley et al. (43), on NSSI measurement challenges). Even so—beyond the specific hypotheses we tested—our up-to-date data demonstrate that NSSI remains both common and clinically significant among young people in Austria.

In summary, our findings position everyday autonomic symptoms as a robust self-reported correlate of NSSI and indicate that such symptoms are associated with both the presence of NSSI and greater NSSI involvement across longer reference periods. A key strength of this study is its large sample spanning early adolescence through emerging adulthood (14–30 years). This breadth, however, may limit generalizability to other populations and complicates direct comparisons with studies that focus exclusively on adolescents or adults. Although this age range spans a key developmental period for NSSI, we did not compare adolescents and young adults. Future studies should examine whether associations with autonomic symptoms differ by age. Several additional limitations warrant note. First, the cross-sectional, online design precludes causal inference; longitudinal studies with clinical validation of NSSI and physiological autonomic measures are needed to determine whether self-reported autonomic symptoms precede, follow, or co-develop with NSSI over time. Second, recruitment relied on a convenience, self-selected sample rather than a stratified, population-representative cohort. Although our 12-month NSSI prevalence roughly aligns with representative surveys (e.g., 4, 6), the low proportion of male participants may restrict conclusions about sex- or gender-specific ANS–NSSI associations (see 44, 45). In addition, no instructed-response attention checks or technical verification checks were included, which limits our ability to identify careless or invalid responding in the online survey. Third, despite controlling for ACEs and perceived stress, some COMPASS 31 items (e.g., “heart flutter,” “sweating”) could reflect general distress rather than specific autonomic dysregulation, potentially inflating the observed associations. Finally, we did not assess co-occurring conditions such as depression, anxiety or PTSD, which can independently alter ANS function and frequently co-occur with NSSI. Notably, ACEs were positively associated with autonomic symptoms in our sample, supporting the relevance of early adversity as a distal factor that may shape later bodily stress-regulation difficulties.

### 4.1 Conclusion

In conclusion, this preregistered cross-sectional study provides large-scale evidence that self-reported autonomic symptom burden is associated with NSSI involvement in German-speaking adolescents and young adults. Higher COMPASS 31 scores were consistently related to lower odds of reporting no NSSI episodes and, for the past month, past year, and lifetime, to higher episode counts. By showing that the ANS–NSSI association is reflected not only in laboratory-based physiological studies but also in everyday bodily symptoms reported by young people, the present findings extend prior work and support the clinical relevance of assessing autonomic symptom burden in the context of NSSI. Importantly, these associations were retained after adjustment for ACEs, perceived stress, medication use, and hormonal contraception, suggesting that everyday autonomic symptoms capture a meaningful dimension of NSSI severity beyond established stress-related covariates.

At the same time, we note that direct NSSI assessment remains essential, and physiological studies are needed to determine how self-reported autonomic symptoms correspond to objective autonomic regulation. Clinically, autonomic symptom burden may nevertheless be useful for case formulation, particularly in young people whose NSSI occurs in the context of bodily distress, stress sensitivity, and emotion-regulation difficulties. The cross-sectional design does not support clinical recommendations for autonomic regulation interventions. However, the present findings suggest that interventions targeting autonomic regulation may represent plausible candidates for future research, including breathing-based approaches, relaxation training, and biofeedback (46, 47). Neuromodulatory approaches such as transcutaneous vagus nerve stimulation may also be relevant in light of emerging work on neural mechanisms and neuromodulation in NSSI (for a review, see 48), but these approaches remain to be tested directly in longitudinal or experimental designs that can determine whether changes in autonomic regulation are associated with changes in NSSI severity.

## Data Availability

The datasets presented in this study can be found in online repositories. The names of the repository/repositories and accession number(s) can be found below: OSF: https://doi.org/10.17605/OSF.IO/NEJPQ.
