# Supplementary material for: Autonomic dysregulation and nonsuicidal self-injury: findings from a cross-sectional online survey
Source: Front Psychiatry. 2026 Jul 27;17:1874303. doi: 10.3389/fpsyt.2026.1874303 (PMC13453698; doi:10.3389/fpsyt.2026.1874303)
Supplement: Supplementary file 1 [file Table1.docx]

Supplementary Material

**Supplementary Table 1. Associations between autonomic symptoms (COMPASS-31) and NSSI frequency without covariates.**

| **Time frame** | **Model component** | **Exponentiated coefficient (95 % CI)** | ***p*** |
| --- | --- | --- | --- |
| Past week | Count (IRR) | 1.02 (0.99 – 1.06) | .17 |
|  | Zero (OR) | 0.94 (0.91 – 0.97) | <.001 |
| Past month | Count (IRR) | 1.05 (1.01 – 1.08) | .010 |
|  | Zero (OR) | 0.93 (0.89 – 0.97) | <.001 |
| Past year | Count (IRR) | 1.06 (1.03 – 1.09) | <.001 |
|  | Zero (OR) | 0.92 (0.89 – 0.95) | <.001 |
| Lifetime | Count (IRR) | 1.07 (1.05 – 1.10) | <.001 |

*Note.* Estimates are exponentiated coefficients from preregistered unadjusted zero-inflated negative-binomial models. IRR = incidence-rate ratio from the count component; OR = odds ratio from the zero-inflation component, where values below 1 indicate lower odds of reporting zero NSSI episodes. CI = confidence interval.

**Supplementary Table 2. Zero-inflated negative-binomial models predicting NSSI frequency with covariates.**

| **Predictor** | **Past week IRR (95 % CI)** | **Past week OR (95 % CI)** | **Past month IRR** | **Past month OR** | **Past year IRR** | **Past year OR** | **Lifetime IRR** | **Lifetime OR** |
| --- | --- | --- | --- | --- | --- | --- | --- | --- |
| COMPASS-31 (per point) | 1.03 (1.00–1.06) | 0.94* (0.89–0.99) | 1.05** (1.02–1.08) | 0.95* (0.92–0.99) | 1.05*** (1.03–1.08) | 0.94** (0.91–0.98) | 1.03*** (1.01–1.05) | 0.90*** (0.86–0.95) |
| Perceived stress (PSS-10) | 1.09*** (1.04–1.15) | 0.99 (0.92–1.09) | 1.10*** (1.06–1.15) | 0.96 (0.88–1.04) | 1.09*** (1.06–1.12) | 0.95 (0.90–1.00) | 0.97* (0.94–1.00) | 0.88*** (0.82–0.93) |
| Childhood trauma (CTQ) | 0.99 (0.96–1.03) | 0.90 (0.79–1.03) | 0.97** (0.95–0.99) | 0.87* (0.77–0.99) | 0.98* (0.96–1.00) | 0.89** (0.83–0.96) | 1.11*** (1.09–1.14) | 1.01 (0.97–1.05) |
| Medication use (yes) | 1.90 (0.83–4.36) | 3.89 (0.85–17.9) | 1.07 (0.53–2.19) | 1.61 (0.57–4.54) | 0.94 (0.55–1.60) | 2.21 (0.71–6.96) | 0.60* (0.40–0.89) | 7.09*** (2.35–21.4) |
| Hormonal contraception (yes) | 0.73 (0.24–2.27) | 0.21 (0.03–1.30) | 1.48 (0.58–3.80) | 0.73 (0.30–1.79) | 1.96* (1.00–3.80) | 0.87 (0.42–1.80) | 2.82*** (1.62–4.89) | 1.08 (0.29–4.11) |

*Note.* Estimates are exponentiated coefficients from covariate-adjusted zero-inflated negative-binomial models. IRR = incidence-rate ratio from the count component; OR = odds ratio from the zero-inflation component, where values below 1 indicate lower odds of reporting zero NSSI episodes. All estimates are adjusted for the covariates shown. CI = confidence interval. *p < .05. **p < .01. ***p < .001.

**Supplementary Table 3. Gender-adjusted zero-inflated negative-binomial models predicting NSSI frequency.**

| **Predictor** | **Past week IRR (95% CI)** | **Past week OR (95% CI)** | **Past month IRR (95% CI)** | **Past month OR (95% CI)** | **Past year IRR (95% CI)** | **Past year OR (95% CI)** | **Lifetime IRR (95% CI)** | **Lifetime OR (95% CI)** |
| --- | --- | --- | --- | --- | --- | --- | --- | --- |
| COMPASS-31 (per point) | 1.04* (1.00–1.07) | 0.97 (0.93–1.01) | 1.05*** (1.02–1.09) | 0.97 (0.94–1.01) | 1.06*** (1.03–1.08) | 0.94** (0.90–0.98) | 1.03*** (1.01–1.04) | 0.90*** (0.86–0.94) |
| Perceived stress (PSS-10) | 1.06* (1.00–1.12) | 0.93 (0.86–1.01) | 1.07** (1.02–1.13) | 0.93* (0.87–0.98) | 1.10*** (1.06–1.14) | 0.96 (0.90–1.01) | 0.97* (0.95–1.00) | 0.89*** (0.84–0.94) |
| Childhood trauma (CTQ) | 0.98 (0.95–1.00) | 0.92* (0.84–1.00) | 0.97*** (0.95–0.99) | 0.91** (0.86–0.96) | 0.99 (0.97–1.01) | 0.87** (0.79–0.96) | 1.11*** (1.09–1.12) | 1.01 (0.98–1.04) |
| Medication use (yes) | 1.69 (0.72–3.97) | 2.69 (0.86–8.47) | 1.05 (0.55–2.00) | 1.57 (0.70–3.52) | 1.14 (0.65–2.00) | 2.97* (1.04–8.50) | 0.57** (0.38–0.85) | 4.43* (1.27–15.37) |
| Hormonal contraception (yes) | 1.18 (0.36–3.90) | 0.69 (0.18–2.63) | 1.86 (0.81–4.28) | 1.18 (0.46–3.05) | 1.60 (0.78–3.26) | 0.64 (0.19–2.15) | 2.84*** (1.76–4.58) | 0.56 (0.19–1.61) |
| Gender: male | 0.39 (0.13–1.18) | 0.13 (0.02–1.03) | 0.53 (0.23–1.23) | 0.27* (0.09–0.75) | 1.75 (0.77–3.95) | 1.89 (0.54–6.62) | 0.88 (0.47–1.63) | 4.11** (1.75–9.64) |
| Gender: diverse | 0.13* (0.02–0.73) | 1.66e-05 (0.00–8.89e+64) | 0.27 (0.07–1.04) | 0.07 (0.00–41.43) | 0.62 (0.20–1.92) | 2.39e-07 (0.00–Inf) | 1.18 (0.42–3.31) | 1.57 (0.33–7.48) |

*Note.* Estimates are exponentiated coefficients from exploratory gender-adjusted zero-inflated negative-binomial models. IRR = incidence-rate ratio from the count component; OR = odds ratio from the zero-inflation component, where values below 1 indicate lower odds of reporting zero NSSI episodes. All estimates are adjusted for COMPASS-31, perceived stress, childhood trauma, medication use, hormonal contraception, and gender. Women were the reference category for gender. CI = confidence interval. Some zero-inflation estimates for the diverse gender category were unstable due to sparse data and should be interpreted cautiously. *p < .05. **p < .01. ***p < .001.
